# Venoarterial extracorporeal membrane oxygenation as bridge support for refractory catecholamine-resistant shock and severe lactic acidosis in a patient with metformin exposure and multifactorial contributors: A case report

**DOI:** 10.62838/jccm-2026-0017

**Published:** 2026-04-30

**Authors:** Ans Alamami, Rabee Tawel, Saleh Mahmoud, Shameel Ahammed, Abdurrahmaan Elbuzidi, Nadir Kharma

**Affiliations:** Department of Critical Care Medicine, Hamad General Hospital, Doha, Qatar

**Keywords:** shock, ECMO, ARDS, lactic acidosis, metformin

## Abstract

A 47-year-old male with type 2 diabetes on metformin and hypertension presented with profound hypoxemia, severe metabolic acidosis (pH unrecordable, lactate 17 mmol/L), and progressive cardiac dysfunction in the setting of presumed sepsis. Despite maximal conventional therapy—including mechanical ventilation, broad-spectrum antimicrobials, and high-dose vasopressors—the patient developed refractory shock and multi-organ dysfunction. Venoarterial extracorporeal membrane oxygenation (VA-ECMO) was initiated on hospital day 2 as hemodynamic bridge support, combined with continuous renal replacement therapy (CRRT). This intervention facilitated stabilization of hemodynamics, correction of acidosis, and improvement in organ function. The patient was successfully decannulated and survived to discharge, though with residual cardiomyopathy. Lactic acidosis in this case was likely multifactorial, with metformin exposure as one potential contributor amid acute kidney injury, hypoperfusion, and possible septic elements. This report describes the use of VA-ECMO as supportive therapy in a complex, refractory critical illness scenario, highlighting the importance of timely multidisciplinary escalation while emphasizing diagnostic challenges in attributing causality and the need for cautious patient selection in such high-risk interventions.

## Introduction

Septic shock, defined according to the Third International Consensus Definitions (Sepsis-3) as a subset of sepsis characterized by persisting hypotension requiring vasopressors to maintain a mean arterial pressure ≥65 mm Hg and serum lactate >2 mmol/L despite adequate fluid resuscitation, remains a leading cause of morbidity and mortality in critically ill patients worldwide [1]. When complicated by acute respiratory distress syndrome (ARDS)—defined by the Berlin criteria as acute-onset hypoxemic respiratory failure (PaO_2_/FiO_2_ ≤300 mm Hg with PEEP ≥5 cm H_2_O) with bilateral opacities not fully explained by effusions, atelectasis, or nodules, and not attributable to cardiac failure [2], along with myocardial dysfunction and profound metabolic acidosis, prognosis deteriorates markedly [3,4].

Severe lactic acidosis in septic shock often arises from multifactorial causes, including tissue hypoperfusion, impaired lactate clearance due to acute kidney injury (AKI), and concurrent factors such as metformin exposure in patients with type 2 diabetes mellitus. Metformin-associated lactic acidosis (MALA) is a rare but recognized complication, with contemporary estimates of incidence generally ranging from <10 to approximately 47 cases per 100,000 patient-years in metformin users, particularly those with renal impairment or precipitating insults like sepsis or dehydration [5,6,7]. The pathophysiology involves metformin inhibition of mitochondrial complex I, which impairs oxidative phosphorylation and augments lactate production, although this effect is typically amplified by coexisting conditions rather than occurring in isolation [8]. Sepsis-induced cardiomyopathy, affecting 20–32% of septic patients, manifests as reversible myocardial depression with reduced ejection fraction and contributes to increased mortality [9,10].

In cases of refractory septic shock with severe myocardial dysfunction unresponsive to fluid resuscitation, high-dose vasopressors/inotropes, and conventional supportive measures, venoarterial extracorporeal membrane oxygenation (VA-ECMO) has been increasingly utilized as a bridge to hemodynamic recovery. VA-ECMO provides both circulatory support and oxygenation, enabling stabilization in patients with profound shock where native cardiac output is insufficient to sustain organ perfusion [11–12]. While evidence remains largely observational and derived from case series and registry data, VA-ECMO is considered in selected adults with septic cardiomyopathy and cardiogenic components of shock, particularly when combined with continuous renal replacement therapy (CRRT) for metabolic correction and toxin clearance [3,13]. CRRT plays a central role in managing severe AKI and acidosis by removing accumulated lactate and, when relevant, metformin [14].

This case report describes a 47-year-old male with type 2 diabetes on metformin who developed refractory septic shock complicated by ARDS, severe lactic acidosis (likely multifactorial with contributions from hypoperfusion, AKI, presumed sepsis, and metformin exposure), and progressive cardiomyopathy. Despite maximal conventional therapy, the patient required VA-ECMO as hemodynamic bridge support to facilitate CRRT and recovery. This presentation is noteworthy because it illustrates the challenges of managing overlapping etiologies of profound lactic acidosis in septic shock and demonstrates the feasibility of early VA-ECMO escalation in a young adult with combined distributive, cardiogenic, and metabolic derangements refractory to standard interventions. The case contributes to the evolving literature on extracorporeal support in complex septic shock phenotypes, where timely multidisciplinary intervention may offer salvage potential in otherwise high-mortality scenarios.

## Case Presentation

A 47-year-old Asian male with type 2 diabetes mellitus (managed with metformin 1000 mg twice daily) and essential hypertension presented to the emergency department with a 5-day history of fever, productive cough, vomiting, and profuse non-bloody diarrhea (~10 episodes/day), accompanied by epigastric pain. There was no recent travel or known toxic ingestion.

On arrival via emergency medical services, he exhibited severe hypoxemia (SpO_2_ 69% on room air), tachypnea (respiratory rate 40 breaths/min), and tachycardia (heart rate 120 beats/min). Blood pressure was 140/80 mmHg. Supplemental oxygen via non-rebreather mask improved SpO_2_ to ~80%; non-invasive ventilation (CPAP: PEEP 8 cmH_2_O, FiO_2_ 60%) further increased SpO_2_ to 97%, though tachypnea persisted. Arterial blood gas on 100% FiO_2_ showed unrecordable pH (extreme acidosis), lactate ~17 mmol/L, PaO_2_ 203 mmHg, PaCO_2_ 25 mmHg, and bicarbonate 2–4 mmol/L. The estimated PaO_2_/FiO_2_ ratio was ~338 mmHg initially on CPAP, consistent with mild acute respiratory distress syndrome (ARDS) per Berlin criteria (acute onset, bilateral opacities, non-cardiogenic). Chest radiograph revealed diffuse haziness without focal consolidation.

Initial point-of-care ultrasound (POCUS) demonstrated preserved left ventricular (LV) contractility with collapsible inferior vena cava and bilateral B-lines, but no effusions. Comprehensive labs showed leukocytosis (WBC 25.6 × 10^3^/μL), elevated procalcitonin (7.05 ng/mL), rising C-reactive protein (from 4.9 to 133.5 mg/L), severe acute kidney injury (creatinine 1140 μmol/L [12.9 mg/dL]), thrombocytopenia (platelets 49 × 10^3^/μL), and markedly elevated cardiac biomarkers (troponin-T from 946 to 9274 ng/L; NT-proBNP 4086 pg/mL). Abdominal CT demonstrated basal lung consolidations, perinephric stranding, and no mesenteric ischemia. ECG showed ST elevation in aVR with diffuse depressions (Table 1).

**Table 1.** Laboratory Data by Hospital Day

**Hospital Day**	**pH**	**HCO_3_ (mmol/L)**	**Lactate (mmol/L)**
Day 1 (03 Sep 2025)	6.810	4.5	17.00
Day 2 (04 Sep 2025)	7.180	10.1	17.00
Day 3 (05 Sep 2025)	7.440	24.5	14.00

The clinical picture was dominated by severe septic shock (Sepsis-3 criteria: vasopressor requirement with lactate >2 mmol/L despite fluids) with suspected pulmonary source, complicated by profound metabolic acidosis (likely multifactorial from hypoperfusion, AKI, presumed sepsis, and metformin exposure) and evolving myocardial dysfunction. Initial management included broad-spectrum antibiotics (piperacillin-tazobactam), aggressive fluid resuscitation, cautious bicarbonate infusion, and intubation for worsening confusion and vomiting. Vasopressor requirements escalated rapidly to high-dose norepinephrine (>0.5 μg/kg/min) combined with vasopressin (0.04 units/min) and dobutamine, yet refractory hypotension (mean arterial pressure <60 mmHg) and persistent severe acidosis (pH 6.8, lactate 17 mmol/L) continued. CRRT was initiated briefly but poorly tolerated due to recurrent hemodynamic instability, necessitating temporary discontinuation. The presumed source of septic shock was pulmonary (community-acquired pneumonia), supported by fever, productive cough, diffuse haziness with basal consolidations on chest radiograph and abdominal CT, and laboratory evidence of systemic infection. However, extensive microbiological investigations, including multiple blood cultures (aerobic and anaerobic), sputum culture, urine culture, and a viral respiratory panel (including influenza, RSV, and SARS-CoV-2), returned negative results. No definitive causative pathogen was isolated, consistent with culture-negative severe sepsis.

Serial POCUS revealed progressive LV apical ballooning and hypokinesis (ejection fraction decreased to 21–25% by day 2), suggestive of sepsis-induced cardiomyopathy (or possible sepsis-related stress cardiomyopathy/Takotsubo-like pattern), though invasive hemodynamic monitoring with pulmonary artery catheter was not performed, precluding formal calculation of cardiac output, systemic vascular resistance, or definitive shock phenotyping. Continuous renal replacement therapy (CRRT) was attempted but was not tolerated due to profound instability (Table 2).

**Table 2.** Echocardiography Data by Hospital Day

**Hospital Day**	**LVEF (BP, %)**	**LVEF AutoEF (BP, %)**	**LVOT VTI (cm)**	**LVOT Vmax (m/s)**	**LVOTd (cm)**	**LVOT PGmax (mmHg)**	**LVSV_ LVOT (mL)**	**LVOT CO (L/min)**	**LVOT CI (L/min/m^2^)**	**LVOT SI (mL/m^2^)**	**TAPSE (cm)**	**RVSP (mmHg)**	**TR Vmax (m/s)**	**ECMO flow–VTI (LPM→cm)**
Day 2	50	50	12.73	0.96	1.9	3.70	37	4.44	2.37	19.8				
Day 3		21.84	6.22	0.60	2.1	1.45	22	2.12	1.13	11.8				3.3→6; 3.0→6.5; 2.5→7
Day 5	24		9.30	0.63	2.0	1.59	28	2.58	1.38	15.0		44.92	2.95	2.5→8.8; 2.0→9.8; 1.5→9.2
Day 6			10.14	0.60		1.47								3.5→7.6; 3.0→9.1; 2.5→9.8; 2.0→11.9; 1.5→11.2
Day 7		26.67	13.45	0.71		2.02								
Day 8	45	46.77	18.52	0.82	2.0	2.72	56	4.63	2.48	29.9	1.8	51.46	3.22	

Given refractory catecholamine-resistant shock, persistent severe lactic acidosis, multi-organ failure, and progressive cardiac dysfunction despite maximal medical therapy, VA-ECMO was initiated on hospital day 2 via femoral cannulation (initial flow 3.5 L/min) as bridge hemodynamic support. This facilitated immediate stabilization, enabling CRRT resumption. Over 4 days of VA-ECMO, hemodynamics improved markedly: lactate decreased to 2.8 mmol/L, bicarbonate rose to 19 mmol/L, creatinine improved to 250 μmol/L [2.8 mg/dL], and vasopressor requirements declined. Ventilatory support was optimized (FiO_2_ 55%, PEEP 12 cmH_2_O; SpO_2_ 99%). Hematologic parameters included hemoglobin drop to 7.8 g/dL (requiring transfusion) with persistent thrombocytopenia. Cardiac biomarkers trended downward, though residual cardiomyopathy persisted (ejection fraction recovered to 45% by discharge). The patient was successfully decannulated after 4 days and survived to hospital discharge with ongoing cardiology follow-up.

## Discussion

This case illustrates the management of refractory septic shock complicated by severe lactic acidosis, ARDS with protective lung ventilation and PEEP optimization [15], acute kidney injury, and progressive myocardial dysfunction in a patient with type 2 diabetes on metformin. The clinical course was dominated by severe sepsis (evidenced by fever, leukocytosis, markedly elevated procalcitonin and CRP, thrombocytopenia, pulmonary consolidations, and multi-organ failure requiring vasopressor support), which fully accounts for the profound lactic acidosis (lactate 17 mmol/L persisting despite resuscitation), shock, and organ dysfunction. Lactic acidosis in septic shock is typically multifactorial, arising primarily from tissue hypoperfusion and impaired clearance due to AKI, with potential amplification by concurrent metformin exposure in the setting of reduced renal function, though plasma metformin levels were not measured, precluding definitive attribution to MALA.

Myocardial dysfunction emerged as a critical component, with serial point-of-care ultrasound demonstrating progressive left ventricular hypokinesis and apical ballooning, reducing ejection fraction to 21–25%. This pattern raised differential considerations including Takotsubo-like stress cardiomyopathy or ischemic injury; however, the clinical context of severe sepsis and subsequent partial recovery strongly favored sepsis-induced cardiomyopathy (SIC) [16]. SIC affects 20–60% of septic patients depending on screening intensity and definitions (typically ejection fraction <45–50%), with pathophysiological mechanisms including cytokine-mediated myocyte depression, nitric oxide excess, mitochondrial impairment, and microcirculatory dysfunction [9,10,11]. Prevalence estimates vary, with prospective cohorts detecting rates up to 60%, often reversible within 7–10 days, though associated with elevated mortality risk (odds ratio 2–3) [17].

This cardiac involvement shifted the shock phenotype from pure distributive to mixed cardiogenic-distributive, explaining catecholamine resistance and initial intolerance to CRRT. The decision to initiate VA-ECMO reflects its evolving role as rescue therapy in adult refractory septic shock with significant myocardial depression [3]. Immediate hemodynamic stabilization with VA-ECMO enabled uninterrupted CRRT, facilitating lactate clearance and metabolic control; emerging data suggest that combined ECMO and CRRT may influence mortality risk in such complex phenotypes [18].

Critiques of SIC diagnosis include diagnostic heterogeneity, over-reliance on ejection fraction without invasive hemodynamics, risks of misclassifying preload or hyperdynamic states, and limited real-world correlation with inflammatory markers, as shown in a prospective study demonstrating no linkage between cytokines and myocardial dysfunction [19]. Unlike pediatric sepsis, where VA-ECMO survival exceeds 70% [20], adult application remains controversial due to concerns of left ventricular distension and differential oxygenation in high-output vasoplegia [12]. Accumulating evidence supports selective use in hypodynamic or cardiomyopathy-dominant subsets, with technical considerations for VA-ECMO in profound cardiogenic shock applicable to septic contexts [21].

The evidence base consists of observational studies, registries, and meta-analyses without randomized trials a key limitation introducing selection bias and center-volume effects. Reported survival varies widely: registry and multicenter data often show 15–36% survival, reflecting real-world challenges [22,23], while selected high-volume series report higher rates with early initiation and reversible cardiomyopathy. Complications including bleeding (30–50%), limb ischemia, and infection further temper enthusiasm, particularly in pure distributive shock [22,23].

Comparatively, our case demonstrates VA-ECMO's bridge utility in enabling CRRT and perfusion restoration [3,14]. Adjunctive strategies (methylene blue, levosimendan) were considered but insufficient amid instability. Survival may reflect patient-specific factors (young age, timely therapy) rather than ECMO alone.

Key limitations include absence of invasive hemodynamics for precise phenotyping; unmeasured metformin levels precluding MALA confirmation; culture-negative sepsis; and single-case design prohibiting causal inference. ECMO risks (bleeding, thrombocytopenia, infection, limb ischemia) highlight the need for high-volume expertise.

This report contributes to the literature on extracorporeal support in complex septic shock, emphasizing cautious selection for VA-ECMO in cases with significant myocardial depression [3]. As evidence evolves, the need for prospective multicenter trials incorporating biomarkers for risk stratification remains pressing. Clinically, it reinforces early SIC recognition, multidisciplinary escalation, and balanced interpretation of multifactorial acidosis in metformin-exposed patients.

## Conclusion

This case report describes the successful use of VA-ECMO combined with CRRT in the management of refractory septic shock complicated by profound multifactorial lactic acidosis with tissue hypoperfusion, acute kidney injury, and metformin exposure
